# The hydrolytic water molecule of Class A β-lactamase relies on the acyl-enzyme intermediate ES* for proper coordination and catalysis

**DOI:** 10.1038/s41598-020-66431-w

**Published:** 2020-06-23

**Authors:** Yunjiao He, Jinping Lei, Xuehua Pan, Xuhui Huang, Yanxiang Zhao

**Affiliations:** 1 The Hong Kong Polytechnic University Shenzhen Research Institute, Shenzhen, P. R. China; 2 Department of Applied Biology and Chemical Technology, State Key Laboratory of Chemical Biology and Drug Discovery, The Hong Kong Polytechnic University, Hung Hom, Kowloon, Hong Kong P. R. China; 3 Department of Chemistry, Center of Systems Biology and Human Health, The Hong Kong University of Science and Technology, Clear Water Bay, Kowloon, Hong Kong P. R. China; 4 School of Pharmaceutical Sciences, Sun Yat-Sen University, Guangzhou, P. R. China

**Keywords:** Biochemistry, Biological techniques, Structural biology

## Abstract

Serine-based β-lactamases of Class A, C and D all rely on a key water molecule to hydrolyze and inactivate β-lactam antibiotics. This process involves two conserved catalytic steps. In the first acylation step, the β-lactam antibiotic forms an acyl-enzyme intermediate (ES*) with the catalytic serine residue. In the second deacylation step, an activated water molecule serves as nucleophile (WAT_Nu) to attack ES* and release the inactivated β-lactam. The coordination and activation of WAT_Nu is not fully understood. Using time-resolved x-ray crystallography and QM/MM simulations, we analyzed three intermediate structures of Class A β-lactamase PenP as it slowly hydrolyzed cephaloridine. WAT_Nu is centrally located in the *apo* structure but becomes slightly displaced away by ES* in the post-acylation structure. In the deacylation structure, WAT_Nu moves back and is positioned along the Bürgi–Dunitz trajectory with favorable energetic profile to attack ES*. Unexpectedly, WAT_Nu is also found to adopt a catalytically incompetent conformation in the deacylation structure forming a hydrogen bond with ES*. Our results reveal that ES* plays a significant role in coordinating and activating WAT_Nu through subtle yet distinct interactions at different stages of the catalytic process. These interactions may serve as potential targets to circumvent β-lactamase-mediated antibiotic resistance.

## Introduction

**Figure 1:**
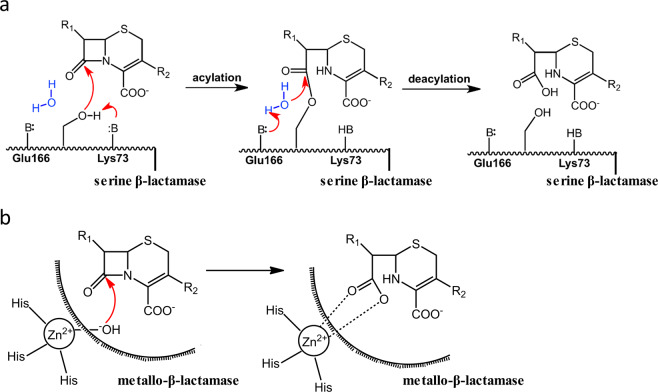
The distinct catalytic mechanisms of β-lactamases from Class A to D. (**a**) Serine-based β-lactamases of Class A, C and D employ a two-step process to hydrolyze β-lactam antibiotics. Key components of the catalytic machinery are labeled, including the catalytic serine residue (Ser), the general base for the acylation step (B_A_) as well as for the deacylation step (B_D_). Amino acid residues fulfilling these roles in Class A are listed as well. (**b**) The catalytic mechanism of Class B metallo-β-lactamases. Only one Zn^2+^ ion is shown for reference while two Zn^2+^ ions have been found in the active site of some Class B metallo-β-lactamases.

Over 1,000 β-lactamases have been reported so far and they are grouped into four classes A to D based on sequence and structural similarities^1,2^. Hydrolysis of β-lactam antibiotics by β-lactamases always involves a critical water molecule that, upon activation, carries out nucleophilic attack on the β-lactam moiety to hydrolyze and “open” its ring structure^3–5^. Class A, C and D β-lactamases are serine-based enzymes that execute the hydrolytic process in two steps (Fig. 1a). At the first acylation step, a conserved catalytic serine residue carries out nucleophilic attack on the β-lactam substrate, leading to the formation of an acyl-enzyme adduct (ES*). At the subsequent deacylation step, an activated water molecule attacks ES*, leading to its hydrolysis and release from the active site with the β-lactam ring “opened”. Two general base residues are required in this process, one to activate the catalytic serine residue in the acylation step, and the other to activate the hydrolytic water molecule in the deacylation step. Extensive studies have identified the individual residues that fulfill these catalytic roles in Class A, C and D β-lactamases. For example, in Class A β-lactamases, Ser70 is the key catalytic residue while Lys73 and Glu166 serve as the general base for acylation and deacylation steps respectively (Fig. 1a)^6^.

In contrast to Class A, C and D, Class B β-lactamases are metallo-enzymes that employ Zn^2+^ or other metal ions to activate a water molecule for direct hydrolysis of the β-lactam substrate without the intermediate step of forming ES* (Fig. 1b)^3,4^. This distinct catalytic mechanism is partly responsible for the extreme promiscuity of Class B β-lactamases. In fact, these enzymes can readily hydrolyze all clinically available β-lactam antibiotics including the newest-generation carbapenems that are usually poor substrates for serine-based β-lactamases of Class A, C and D^7,8^.

While many aspects of the catalytic process for serine-based Class A, C and D β-lactamases have been well characterized, structural factors that coordinate and activate the critical water molecule are not fully understood. In particular, many crystal structures have captured reaction intermediates of the active site when catalytic activity is stalled by mutating key residues or using non-hydrolyzable transition analogs^9–12^. As a result, the water molecules identified in these structures may not represent the catalytically competent state for nucleophilic attack.

To capture the critical hydrolytic water molecule undergoing active catalysis in serine-based β-lactamases, we made a mutant Class A β-lactamase substituting Glu166, the general base for deacylation, with tyrosine. This Glu166Tyr mutant showed sufficient residual activity that allowed us to track the hydrolytic water molecule as the active site underwent a full cycle of *in crystallo* catalysis. Our crystallographic studies of Glu166Tyr led to identification of previously underappreciated structural and environmental factors in the active site that work synergistically to coordinate and activate the hydrolytic water molecule for nucleophilic attack on ES*.

## Results

### Glu166Tyr mutation in Class A β-lactamase PenP leads to moderately slowed kinetic rate for cephaloridine hydrolysis

Our lab has used PenP from *Bacillus licheniformis* as a model Class A β-lactamase in a series of studies to delineate its catalytic mechanism^13–15^. A recent study by Stojanoski et. al. reported that substituting the general base Glu166 with Tyr in TEM-1 led to reduced but still measurable activity toward most β-lactam antibiotics and unexpectedly caused higher hydrolytic activity for ceftazidime, a third-generation cephalosporin with bulky branched side chain^16^. We hypothesize that incorporating the same Glu166Tyr mutation in PenP may lead to similar kinetic rate so that we can track the coordination and activation of the catalytic water molecule as the active site undergoes a full cycle of catalysis.

**Figure 2:**
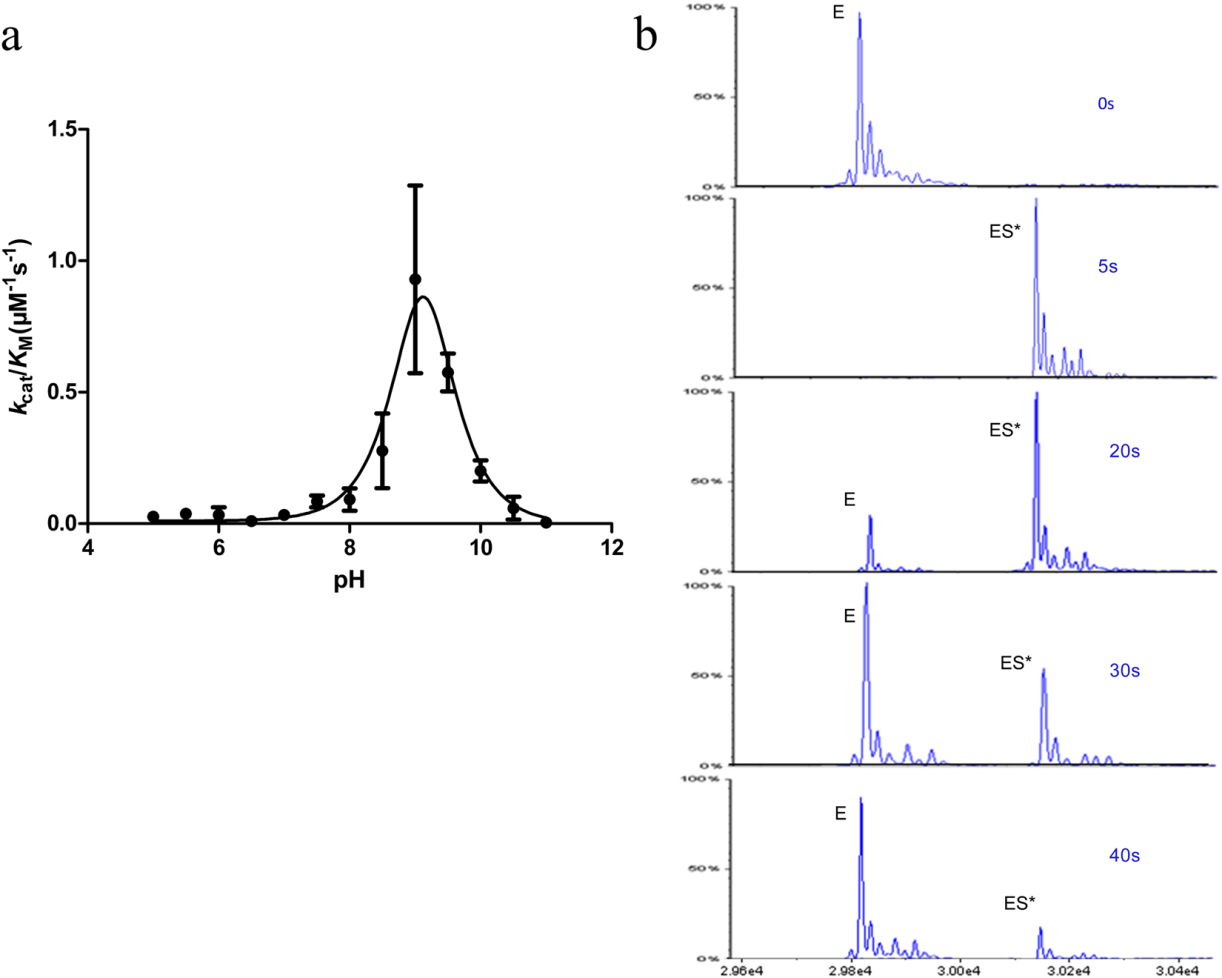
Kinetic profile of Class A β-lactamase PenP with Glu166Tyr mutation. (**a**) The pH-dependent *k*_*cat*_*/K*_*M*_ profile of PenP Glu166Tyr mutant measured by nitrocefin assay. (**b**) The time-dependent ESI-MS profile of PenP Glu166Tyr hydrolyzing cephaloridine reveals the moderately slowed hydrolytic process. E: the free enzyme (29,821 Da). ES*: the enzyme-substrate acyl adduct (30,157 Da).

We characterized the kinetic profile of PenP Glu166Tyr mutant by UV-visible spectroscopy and electrospray ionization mass spectrometry (ESI-MS). The reduction in overall catalytic efficiency (*k*_*cat*_/K_M_) for Glu166Tyr is substrate-dependent, with ~1,000 fold reduction for penicillin G but only ~20 fold for another β-lactam cephaloridine (Table 1). Furthermore, the pH profile for Glu166Tyr showed a significant shift toward the basic region with maximum enzyme activity at ~pH 9.5 while the optimal pH for wild-type PenP was at ~pH 7.0 (Fig. 2a). This shift is likely due to the different titration profile of Tyr166 when it replaces Glu166 as the general base in the catalytic process. Lastly, ESI-MS profile showed that the deacylation reaction for Glu166Tyr was reasonably fast with the ES* adduct fully hydrolyzed and converted back to free enzyme (E) after only ~40 seconds (Fig. 2b). Based on these observations, we decided to conduct time-dependent x-ray crystallography studies of Glu166Tyr mutant undergoing *in crystallo* hydrolysis of cephaloridine to identify factors critical for coordinating and activating the hydrolytic water molecule.

**Table 1:** Kinetics profile for Glu166Tyr.

β-lactamase	antibiotics	K_m_(μM)	k_cat_(s^−1^)	k_cat_/K_m_(M^−1^s^−1^)
WT	Penicillin G	60.9 ± 5.3	393.8 ± 18.5	(6.5 ± 0.9) × 10^6^
	cephaloridine	20.7 ± 4.1	8.2 ± 0.8	(4.0 ± 1.0) × 10^5^
E166Y	Penicillin G	196.5 ± 47.5	0.81 ± 0.08	(4.1 ± 1.1) × 10^3^
	cephaloridine	3.0 ± 0.4	0.055 ± 0.002	(1.8 ± 0.3) × 10^4^

### The *apo* structure of Glu166Tyr reveals a centrally positioned water molecule (WAT_Nu) coordinated by a network of hydrogen bonds

**Figure 3:**
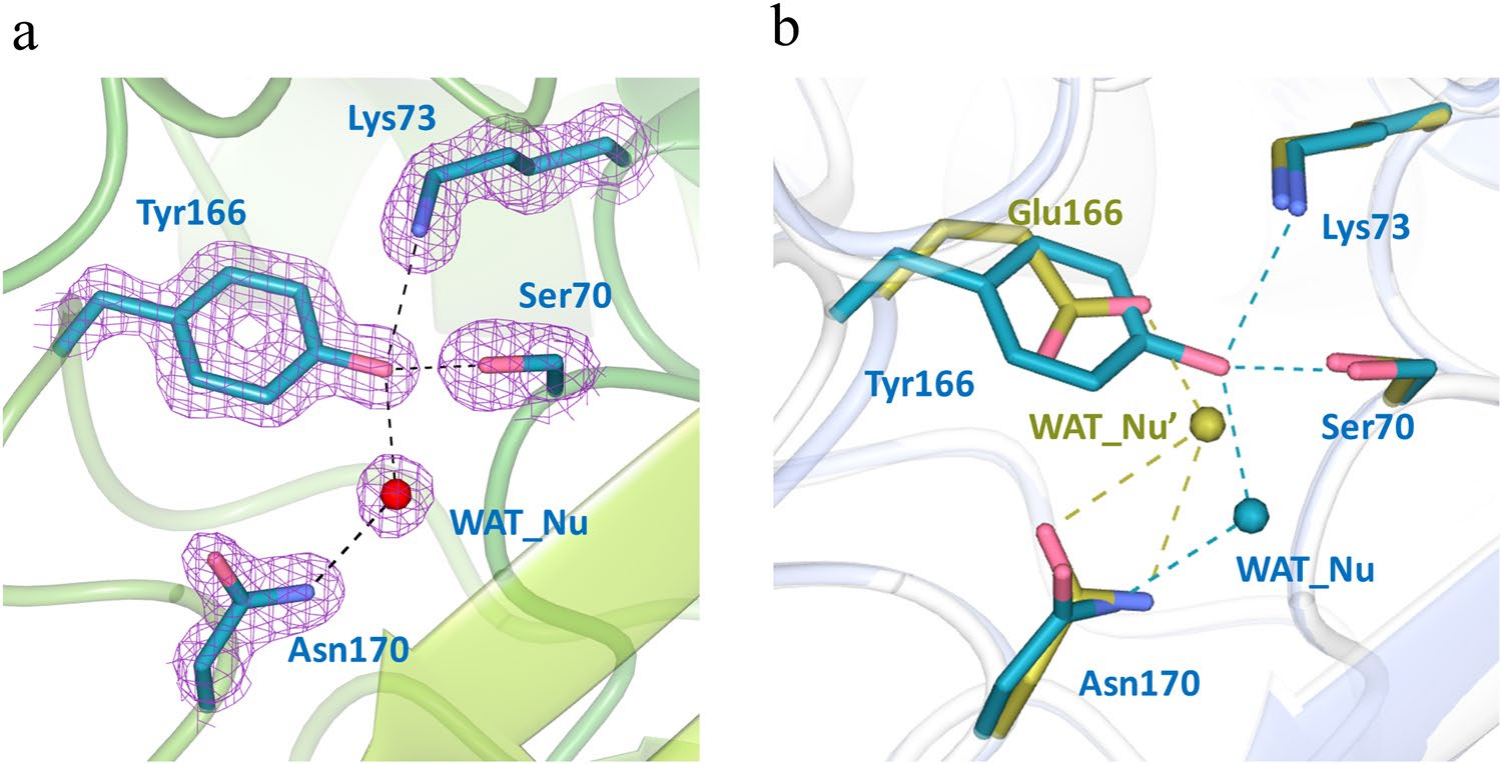
The *apo* structure of Glu166Tyr reveals an extensive hydrogen bonding network for Tyr166 and a centrally located water molecule in the active site. (**a**) Tyr166 forms a network of hydrogen bonds with other key catalytic residues and a hydrolytic water molecule (WAT_Nu) in the active site. *Fo-Fc* omit map (purple) is drawn in mesh format and contoured at 2.0 σ. The dashed lines indicate hydrogen bonds. (**b**) Superposition of Glu166Tyr active site (cyan) with that of the wild-type (golden). WAT_Nu: the putative hydrolytic water molecule in the wild-type structure. Figures are made using CCP4mg.

A series of Glu166Tyr structures reflecting distinct reaction intermediates were obtained at high resolutions of 1.5–2.0 Å by x-ray crystallography (Table 2). The overall structure of PenP is nearly identical in all these structures, with Root Mean Square Deviation (RMSD) of only ~0.3 Å as compared to the wild-type. In the active site of the *apo* structure, the mutated residue Tyr166 adopts the same conformation as the Glu166 residue in the wild-type structure but the bulky side chain of Tyr166 extends much closer toward the catalytic residue Ser70 and forms a stable hydrogen bond with it (distance ~2.8 Å) (Fig. 3a,b). The Glu166Tyr mutation has little impact on the overall structure of the active site as nearby residues like Ser70, Lys73 and Asn170 adopt essentially identical conformation as in wild-type structure (Fig. 3b).

**Table 2:** Statistics of x-ray crystallography data collection and structure refinement.

PDB ID	*apo*	Post-acylation	Deacylation-3s
	5ZFL	5ZG6	5ZFT
***Data collection***			
Space group	P1	P1	P1
**Unit cell parameters**			
*a, b, c* (Å)	43.23, 48.40, 66.21	43.29, 46.46, 65.98	43.29, 45.49, 66.10
*α, β, γ* (^°^)	76.29, 75.38, 69.56	77.43, 75.53, 69.34	77.81, 75.48, 68.76
Resolution range(Å)	63.20-1.50 (1.53-1.50)	63.20-2.00 (2.03-2.00)	42.02-1.93 (2.03-1.93)
No. of total reflections	139535	97356	121668
**No. of unique reflections**	72109	29472	31135
*I/σ*	19.8 (2.3)	16.3 (4.5)	12.2(5.6)
Completeness(%)	93.9 (82.2)	94.0 (83.9)	91.2 (86.3)
*R*_merge_(%)	5.6 (31.5)	11.3 (31.3)	5.5 (12.8)
***Structure refinement***			
Resolution(Å)	63.20-1.50	43.06-2.00	35.10-1.93
*R*_cryst_/*R*_free_(%)	16.9/20.8	20.9/25.9	17.8/22.3
r.m.s.d. bonds(Å)/angles(^°^)	0.014/1.671	0.017/1.930	0.019/1.943
**No. of reflections**			
Working set	68457	27895	30373
Test set	3633	1486	1624
*No. of atoms*			
Protein atoms	4166	4012	4174
Ligand/ion atoms	68	44	44
Water molecules	542	104	130
Average *B*-factor(Å^2^)			
Main chain	16.896	16.883	12.360
Side chain	23.372	19.438	16.159

Notably, the key catalytic water molecule, termed WAT_Nu for its potential role as the hydrolytic nucleophile, forms totally six hydrogen bonds with partners including the side chains of Ser70, Tyr166 and Asn170 as well as the mainchain carbonyl of Ala237, the main chain amide of Ser70 plus a possible sulfate ion from the bulk solvent (Fig. 3a,b and Table 3). Compared to a similar water molecule observed in the wild-type TEM-1 structure, WAT_Nu in Glu166Tyr mutant structure is located closer to Ser70 and Asn170, but farther away from Lys73 (Fig. 3b). This difference is probably due to the bulkier side of Tyr166 as compared to Glu166, resulting in WAT_Nu being “pushed” away from Lys73 and toward Ser70 instead.

**Table 3:** Hydrogen bonding network for WAT_Nu. Notably changes compared to the *apo* structure are underlined and in bold font.

Hydrogen bond distances (Å)	Apo	Post-acylation	Deacylation-3s-attack	Deacylation-3s-tiled
WAT_Nu – Ccarbonyl of acyl adduct	—	3.04	***2.28***	3.04
WAT_Nu – Ser70 Oγ	2.93	***3.95***	3.39	3.18
WAT_Nu – Tyr166 Oη	2.83	***2.47***	2.73	2.38
WAT_Nu – Asn170 Nδ	2.63	***2.28***	2.22	2.31
Ser70 Oγ – Lys73 Nζ	2.83	***3.54***	3.10	3.25
Ocarbonyl of acyl adduct – Ser70 N	—	3.00	3.21	***2.67***
Ocarbonyl of acyl adduct – Ala237 N	—	2.68	2.65	***3.10***

### The post-acylation structure shows subtle displacement of WAT_Nu and Tyr166 by the newly formed ES* acyl adduct

**Figure 4:**
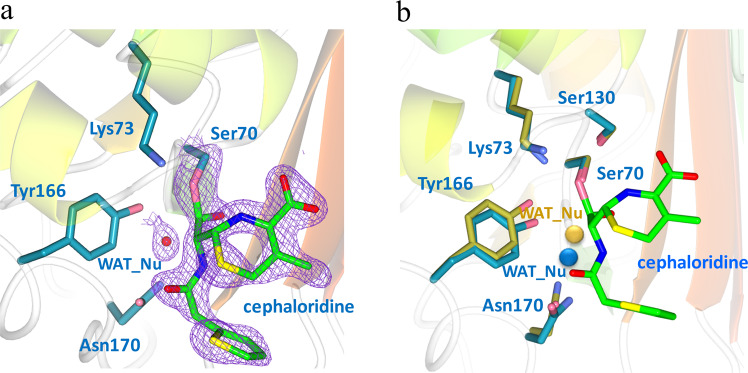
The post-acylation structure of Glu166Tyr shows displacement of Tyr166 and DW away from the PenP-cephaloridine acyl adduct. (**a**) The active site in the post-acylation structure. *Fo-Fc* omit map (purple) is drawn in mesh format and contoured at 2.0 σ to highlight the newly formed acyl adduct between Ser70 and the cephaloridine substrate. The dashed lines indicate hydrogen bonds. (**b**) Superposition of post-acylation structure (cyan) with the *apo* structure (golden). The acyl adduct is highlighted in green. The two water molecules are enlarged for presentation effect.

The post-acylation structure was obtained when crystals of Glu166Tyr were soaked in cephaloridine-containing buffer for 7 minutes and then directly mounted onto x-ray machine for data collection. In this structure, the ES* adduct with covalent linkage between cephaloridine and Ser70 is clearly visible in the *fo-fc* map (Fig. 4a). The conformation of ES* is nearly identical to that seen in our previously reported PenP Glu166His structure as well as in other Class A β-lactamases like PC1^14,17–19^.

However, the newly formed ES* adduct actually impinges onto the position occupied by WAT_Nu in the *apo* structure (Fig. 4b). As a result, WAT_Nu is displaced away from Ser70 by ~1.2 Å. Glu166Tyr and Asn170 are also displaced in the same direction by 1.05 Å and 0.95 Å respectively (Fig. 4b). After these subtle movements, WAT_Nu is ~2.9 Å away from the ES* adduct but still retains hydrogen bonds with Glu166Tyr, Asn170 and Ala237 (Table 3).

### Two novel conformations in the deacylation-3s structure delineate critical factors to activate WAT_Nu for nucleophilic attack on acyl adduct

**Figure 5:**
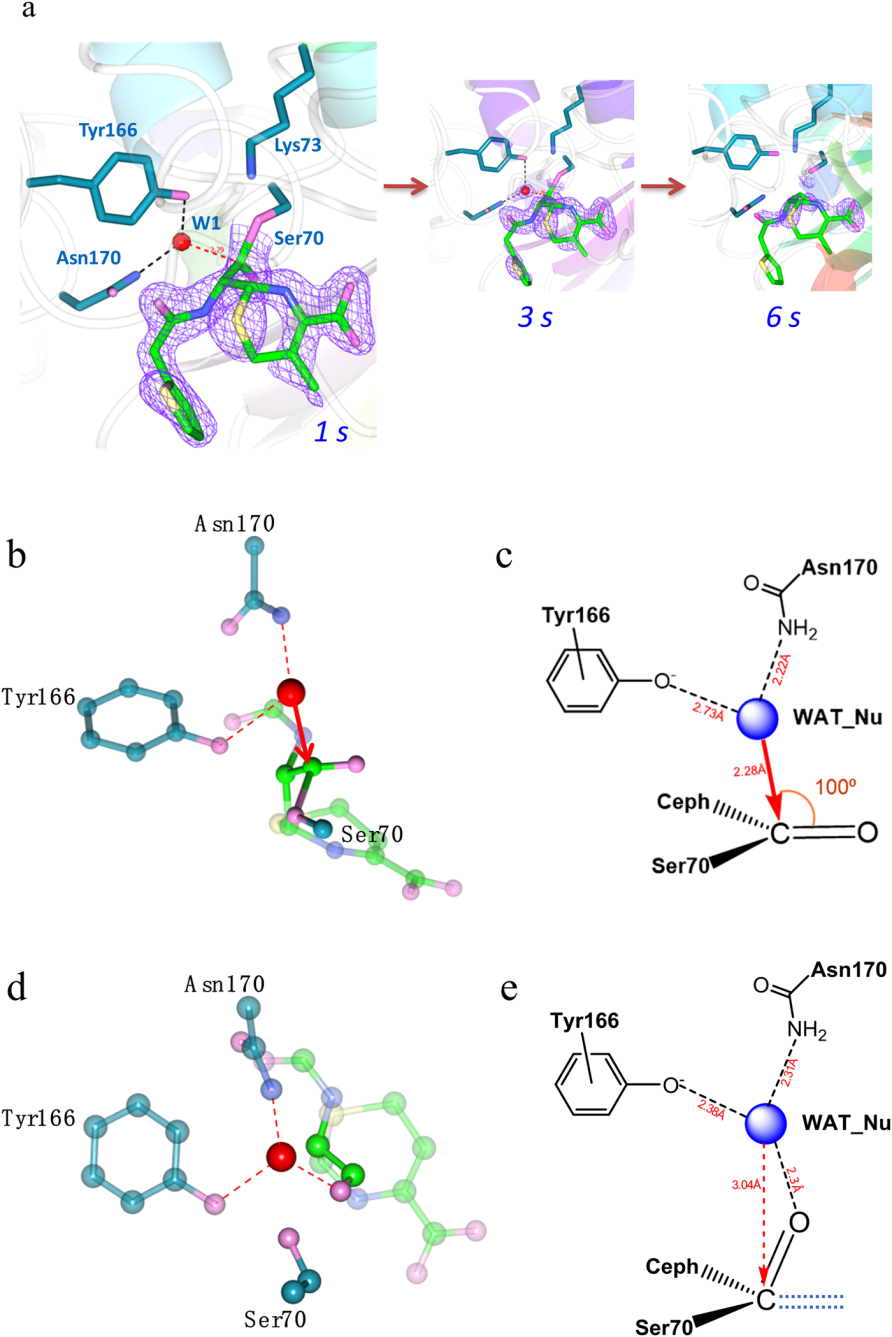
Structures of deacylation intermediates reveal critical factors to activate the catalytic water molecule. (**a**) The time-dependent profile of *in crystallo* deacylation process as depicted by three structures captured at 1, 3 and 6 seconds respectively. The *Fo-Fc* omit map (purple) is drawn in mesh format and contoured at 2.0 σ to highlight the disappearance of the acyl adduct. (**b**) The catalytically competent conformation of the deacylation-3s structure shows WAT_Nu poised with perfect geometry to carry out nucleophilic attack on the acyl adduct. (**c**) Scheme presentation of the Bürgi-Dunitz trajectory for this conformation. (**d**) The catalytically incompetent conformation of the deacylation-3s structure reveals a hydrogen bond between WAT_Nu and the “tilted” carbonyl oxygen of the acyl adduct. (**e**) Scheme presentation of (**d**). The dashed lines represent the orientation of the carbonyl oxygen in the catalytically competent conformation in (**c**).

We set out to capture structures for *in crystallo* deacylation reaction intermediate by transferring cephaloridine-soaked Glu166Tyr crystals into cephaloridine-free buffer for 1, 3 or 6 seconds and then flash-freezing for x-ray data collection. The short equilibration time was set to match the relatively fast deacylation rate of Glu166Tyr mutant. The resulting structures are termed deacylation-1s, −3s and −6s accordingly. The ES* adduct was clearly visible in the deacylation-1s structure but largely disappeared in the deacylation-6s structure, suggesting active *in crystallo* hydrolysis (Fig. 5a). Such pattern of change is highly similar to what we observed in a previous study of PenP Glu166His mutant, but occurs over a much shorter period (6 seconds vs. 3 minutes)^14^.

Notably, the deacylation_3s structure reveals two distinct conformations in the active site. In one of the two Glu166Tyr molecules in the asymmetric unit, both WAT_Nu and Glu166Tyr are shifted back toward ES*, occupying positions similar to those observed in the *apo* structure (Fig. 5b). Under this new conformation, WAT_Nu re-establishes hydrogen bond with Ser70 and is positioned within ~2.3 Å away from the carbonyl carbon of ES*on the right trajectory for nucleophilic attack. In this “attack” conformation, the Bürgi–Dunitz (BD) angle of the approaching trajectory, i.e. defined as the WAT_Nu-C-O angle, is ~100°. The Flippin–Lodge (FL) angle that describes the offset of the approaching trajectory relative to the carbonyl of ES*, is ~0° as WAT_Nu is positioned almost directly behind ES*with symmetric arrangement relative to the carbonyl (Fig. 5c and Table 3). Both values agree with the BD and FL angles predicted by organic chemistry principles and observed in other serine-based enzymes such as elastase and subtilisin^20,21^. This is the first time the catalytic water molecule of Class A β-lactamase is visualized near the Bürgi-Dunitz trajectory poised for nucleophilic attack on the carbonyl of ES*.

Unexpectedly, for the other Glu166Tyr molecule in the asymmetric unit, an alternative conformation with the carbonyl of ES* tilted almost 90° upward and away from the amide group of Ala237 of the oxyanion hole (Fig. 5d). In this “tilted” conformation, WAT_Nu forms a strong hydrogen bond directly with the carbonyl oxygen of ES* across a short distance of only ~2.3 Å, locking itself into a conformation not suitable for nucleophilic attack (Fig. 5e and Table 3). It is intriguing that such a subtle movement by ES* renders catalysis not possible even though WAT_Nu is retained in close proximity.

### QM/MM calculations confirm the catalytic status of the two novel conformations with one poised for nucleophilic attack and the other catalytically incompetent

Our deacylation-3s structure revealed two novel conformations in the active site of Glu166Tyr, with the “attack” conformation showing WAT_Nu positioned with perfect geometry on trajectory for nucleophilic attack and the other “tilted” conformation showing WAT_Nu trapped in a catalytically incompetent conformation. To confirm the catalytic status of these two conformations, we conducted QM/MM calculations to model the WAT_Nu-mediated hydrolytic process in each structure.

**Figure 6:**
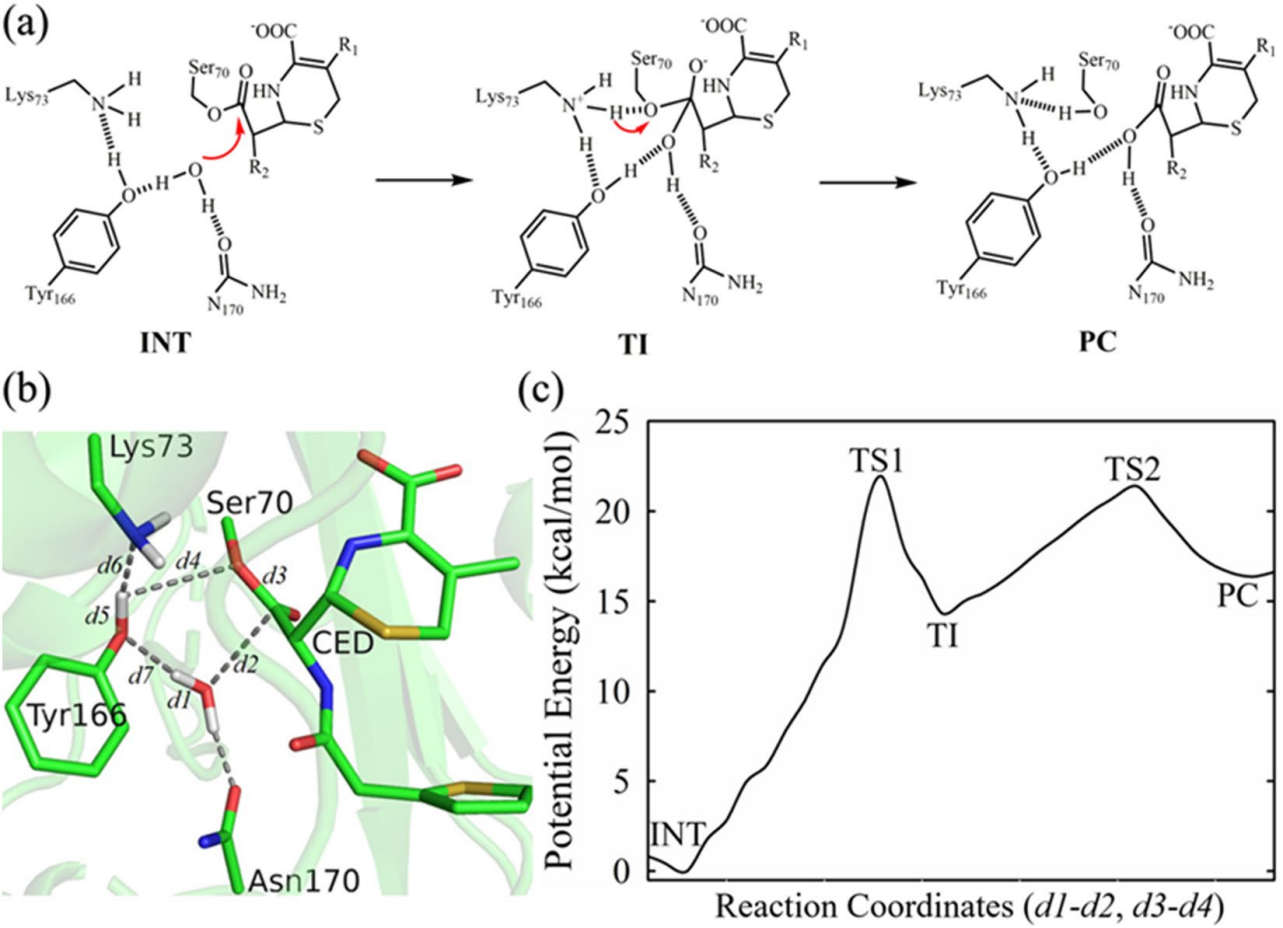
QM/MM calculations of the deacylation-3s structure confirm the “attack” conformation as energetically favorable. (**a**) The reaction scheme for the deacylation step of the “attack” conformation according to the prevailing model. INT: acyl adduct intermediate. TI: tetrahedron intermediate. PC: product complex. (**b**) Schematics of the sub-system for QM/MM analysis of the “attack” conformation. CED: cephaloridine. Various distances in the active sites are labeled as *d*1 to *d*7. Figure is prepared using PyMOL2.3 (https://pymol.org/2/) (**c**) One dimensional potential energy profile for the deacylation pathway starting from the “attack” conformation. It was obtained as the minimal potential energy pathway identified by Dijkstra’s algorithm from the two-dimensional potential energy surface with reaction coordinates: RC = *d1*-*d2* and *d3*-*d4* (see Experimental Procedures for details). In particular, reaction coordinate *d3*-*d4* tracks the breaking of C-O bond between ligand CED and Ser70 (*d3*), and the proton transfer from Tyr166 to Ser70 (*d4*). Reaction coordinate *d1*-*d2* tracks the nucleophilic attack of deacylation water WAT_Nu to the carbonyl carbon of ligand CED (*d2*), and the proton leaving from deacylation water WAT_Nu (*d1*). The potential energy barrier to transit from INT to PC is ~22.8 kcal/mol. TS1 and TS2: transition state 1 and 2. The potential energies for INT, TS1, TI, TS2, PC are 0, 22.79 ± 1.37, 16.76 ± 1.38, 22.59 ± 2.06, 18.07 ± 1.68 kcal/mol, respectively.

For the deacylation reaction, the prevailing model in the literature states that Glu166, i.e. Tyr166 in our mutant structure, serves as general base to activate WAT_Nu by proton abstraction and the activated WAT_Nu carries out subsequent nucleophilic attack on ES*, resulting in its hydrolysis and release of the ligand (Fig. 6a).

This prevailing model can be readily applied to the “attack” conformation as WAT_Nu is positioned close to Tyr166 and on the right trajectory for nucleophilic attack on ES*. So for the “attack” conformation, we set up a QM sub-system consisting of deprotonated Lys73, protonated Tyr166, WAT_Nu and ES* to present the active site at the state after the completion of the acylation reaction and yet before the commencement of the deacylation step (Fig. 6b). The assignment of the protonation states for Lys73 and Tyr166 is based on the prevailing model that Lys73 and Glu166, i.e. Tyr166 in our mutant structure, act as general base for the acylation and deacylation steps respectively^3,22–24^. As general base for the acylation step, Lys73 is expected to be deprotonated at the start and end of the acylation step. A proton transfer between deprotonated Lys73 and protonated Tyr166 would then enable Tyr166 to serve as general base for the deacylation step, to activate WAT_Nu by proton abstraction and trigger subsequent nucleophilic attack on ES*.

Two reaction coordinates were set up to track the activation of WAT_Nu by Tyr166 and the subsequent hydrolysis of ES* (Fig. 6b,c). Reaction coordinate *d1-d2* considers the nucleophilic attack of deacylation water WAT_Nu on the carbonyl carbon of ligand CED (*d2*) and the proton leaving from deacylation water WAT_Nu (*d1*). Reaction coordinate *d*3*-d4* describes the breaking of the C-O bond between ligand CED and Ser70 (*d3*) and the proton transfer from Tyr166 to Ser70 (*d4*). Our QM/MM calculations reveal that the deprotonation and nucleophilic attack of WAT_Nu result in the formation of a metastable tetrahedron intermediate (TI), which can be stabilized by extensive hydrogen bond network. Additionally, the tetrahedron intermediate is readily decayed to release product (PC) (Fig. 6c and Supplemental Fig. S1). The potential energy profiles computed from our QM/MM simulations reveal a favorable potential energy barrier of about ~22.8 kcal/mol with good convergence (Fig. 6c and Supplemental Fig. S2). This value is in good agreement with the energy barrier of ~19 kcal/mol calculated from our measured *K*_*cat*_ value of 0.055 s^−1^ (Table 1).

**Figure 7:**
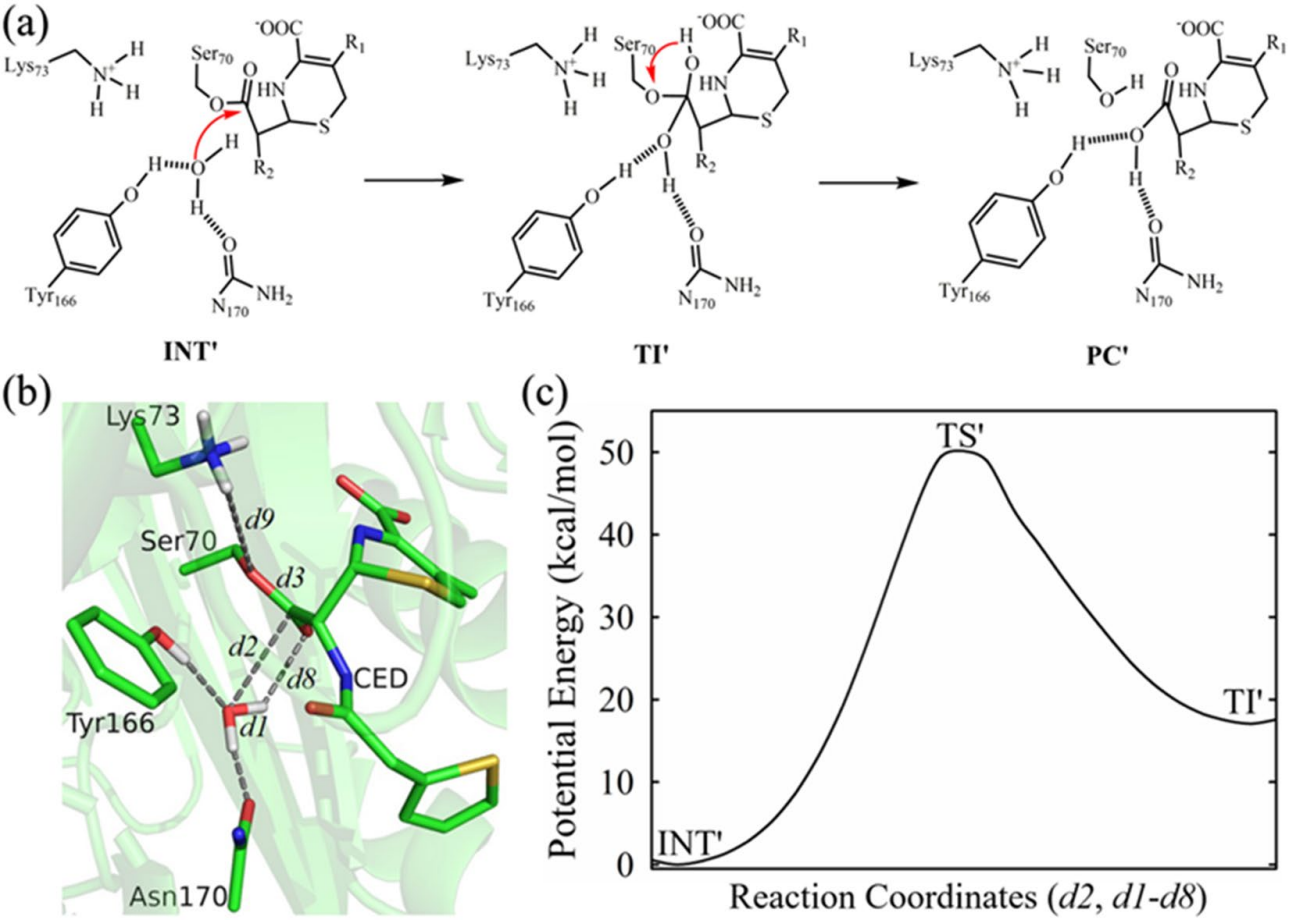
QM/MM analysis of the deacylation-3s structure confirms the “tilted” conformation as energetically unfavorable. **(a**) The reaction scheme for the deacylation step of the “tilted” conformation according to the alternative model. INT’: acyl adduct intermediate. TI’: tetrahedron intermediate. PC’: product complex. These states are marked with’ to differentiate from those in the “attack” conformation. (**b**) Schematics of the sub-system for QM/MM analysis of the “tilted” conformation. CED: cephaloridine. Various distances in the active sites are labelled as *d*1 to *d*9. Figure is prepared using PyMOL2.3 (https://pymol.org/2/) (**c**) One dimensional potential energy profile for the deacylation pathway starting from the “tilted” conformation. It was obtained as the minimal potential energy pathway identified by Dijkstra’s algorithm from the two-dimensional potential energy surface with reaction coordinates: RC = *d2* and *d1*-*d8* (see Experimental Procedures for details). Reaction coordinate *d1*-*d8* denotes the proton transfer from deacylation water WAT_Nu (*d1*) to the carbonyl oxygen of ligand CED (*d8*). Reaction coordinate *d2* tracks the nucleophilic attack of deacylation water WAT_Nu to the carbonyl carbon of ligand CED (*d2*). The potential energy barrier to transit from INT’ to TS’ is high at over ~50 kcal/mol. TS’: transition state. The potential energies for INT’, TS’, TI’ are 0, 49.72 ± 0.33, 17.49 ± 0.41 kcal/mol, respectively.

For the “tilted” conformation, given the unexpected strong hydrogen bond between WAT_Nu and the carbonyl oxygen of the “tilted” ES*, the prevailing model of Tyr166 serving as general base to activate WAT_Nu cannot be applied. Instead we wondered if an alternative substrate-assisted deacylation pathway is possible, with WAT_Nu directly transferring a proton to ES* to initiate subsequent hydrolysis (Fig. 7a).

To test this alternative reaction scheme, we conducted QM/MM calculations with a QM sub-system consisting of only Ser70, WAT_Nu and ES* (Fig. 7a). Two reaction coordinates were set up to check the energetic profile of this alternative scheme (Fig. 7b,c). Reaction coordinate *d1-d8* denotes the proton transfer from deacylation water WAT_Nu (*d1*) to the carbonyl oxygen of ligand CED (*d8*). Reaction coordinate *d2* refers to the nucleophilic attack of deacylation water WAT_Nu on the carbonyl carbon of ligand CED. Our QM/MM calculations reached convergence and reveal that such a scenario is energetically unfavorable because it has an energy barrier of ~50 kcal/mol, significantly higher than that observed in the “attack” conformation (Fig. 7c, Supplemental Fig. S2).

In summary, our QM/MM results suggest that the “attack” conformation is catalytically competent with favorable potential energy profile that matches experimental data. In contrast, the “tilted” conformation with strong hydrogen bond between WAT_Nu and the tilted carbonyl oxygen is not catalytically competent.

## Discussions

β-lactamases inactivate β-lactam antibiotics by hydrolyzing and “opening” their signature β-lactam ring. In serine-based β-lactamases of Class A, C and D, this hydrolytic process is executed in the deacylation step involving an activated water molecule carrying out nucleophilic attack on the enzyme-substrate acyl adduct ES*. Despite extensive studies, active site features that are critical for coordination and activation of this critical water nucleophile are not fully understood.

Here we report structural and computational studies of Class A β-lactamase PenP with Glu166Tyr mutation that analyze the interactions between this critical water molecule, termed WAT_Nu, and other components of the active site during a full cycle of hydrolysis. Our results reveal that, similar to previous reports, active site residues Glu166Tyr, Asn170 and Ala237 play key roles in coordinating WAT_Nu by forming a network of hydrogen bonds as observed in *apo*, post-acylation and deacylation-3s steps. Intriguingly, amino acid substitutions at Asn170 and Ala237 have been reported to lead to expanded substrate profile and antibiotic resistance for β-lactamases. For example, N170S in GES-5 converts this Class A β-lactamase into a carbapenemase^25^. A237G, A237T and G238S in TEM-1 all lead to extended-spectrum β-lactamases (ESBLs) that hydrolyze newer-generation oxyimino-cephalosporins like ceftazidime and cefotaxime^26^. The mechanism of how these mutations expand substrate profile is not well understood. The novel roles for these two residues in coordinating the hydrolytic WAT_Nu as uncovered by our study may offer new perspectives.

Additionally, our study also uncovers previously underappreciated role of ES* in coordinating and activating WAT_Nu during the catalytic process. In the *apo* structure with ES* absent, WAT_Nu is coordinated by a network of hydrogen bonds involving Glu166Tyr, Asn170 and Ala237. However, in the post-acylation structure, the newly formed ES* pushes WAT_Nu away from the catalytic residue Ser70 and closer toward the general base Glu166Tyr. In the subsequent deacylation-3s structure, the activated WAT_Nu moves back toward ES* and becomes positioned near Bürgi-Dunitz trajectory with optimal geometry to carry out nucleophilic attack. Unexpectedly, subtle “tilting” by the carbonyl oxygen of ES* leads to improper hydrogen bond with WAT_Nu and renders the active site catalytically incompetent. Interestingly, such “tilted” or “flipped” conformations for ES* have been reported in several Class A or C β-lactamases like TEM-1, AmpC, and SHV-1 when the active site is inhibited by slowly hydrolyzing carbapenems^27–29^. It is possible that the conformational flexibility of ES* affects the coordination and activation of the catalytic water molecule.

In summary, by analyzing the reaction intermediates observed in the active site of a Class A β-lactamase hydrolyzing a β-lactam substrate, our study has uncovered critical features within the active site that coordinate and activate the water molecule for hydrolysis. In particular, our results uncover subtle yet distinct interactions between the catalytic water molecule and ES* at different stages of catalysis to ensure proper catalysis. Targeting these interactions may provide a potential strategy to circumvent β-lactamase-mediated antibiotic resistance.

### Experimental procedures

#### Protein expression and purification

Wild-type (WT) PenP was subcloned into a modified pET 30a vector containing an N-terminal His6 tag and the human rhinovirus (HRV) 3C protease cleavage site. Glu166Tyr mutant was generated by site-directed mutagenesis (FINNZYME). Protein expression for the WT PenP and Glu166Tyr mutant were done using *E. coli* strain BL21(DE3) following standard procedure. Briefly, inoculated bacteria culture was grown at 37 °C until the OD600 reached 0.6–0.8, then protein expression was induced by adding IPTG at the final concentration of 500 μM. The bacteria culture was grown at 30 °C for an additional 5 hours and then collected by centrifugation. The His6-tagged PenP proteins were purified by HisTrap affinity column (GE Healthcare) and then protease 3C was used to cleave the tag. The target proteins were further purified by gel filtration chromatography (Superdex 75, GE Healthcare) in a buffer of 20 mM Tris, pH 7.5 and 50 mM NaCl. The desired fractions were collected and concentrated by AMICON Ultra-15 Centrifugal Filter Devices (Millipore NMWL = 10000).

#### ESI-MS measurements for enzyme kinetics

The enzyme-substrate binding interaction was initiated by mixing 35 μL of 5 μM PenP Glu166Tyr in 20 mM ammonium acetate (pH 7.0) with 35 μL of 10 μM cephaloridine in the same buffer. At desired time intervals, the reaction was quenched by addition of 70 μL of 1% formic acid (v/v) in acetonitrile. The resulting solution was characterized by electrospray ionization mass spectrometry (ESI-MS). Normally, there are two major peaks in the mass spectrum, one for the enzyme itself (E), the other for the acyl-adduct (ES*). The relative concentration of free enzyme ([E]) and the acyl-adduct ([ES*]) can be determined by integrating the area under measuring the intensity of these two peaks, while the total amount of enzyme ([E_total_]) can be calculated from the sum of [E] and [ES*] ([E_total_] = [E] + [ES*]).The deacylation constant (*k*_*3*_) then can be determined by fitting the value of [ES*]/[E_total_] versus time (t) to Eq. 1.

1 $$[{\rm{E}}{\rm{S}}\ast ]/[{{\rm{E}}}_{{\rm{t}}{\rm{o}}{\rm{t}}{\rm{a}}{\rm{l}}}]=\exp ({k}_{3}t)$$

#### UV spectroscopy measurements for enzyme kinetics

Two antibiotics, penicillin G and cephaloridine, were used in this study, their corresponding wavelength and extinction coefficient was: penicillin G, 232 nm, Δε = 1100 M^−1^cm^−1^; cephaloridine, 260 nm, Δε = 10,200 M^−1^cm^−1^. A brief procedure is as follows. The reaction was started by mixing the substrate and protein in a buffer of 50 mM potassium phosphate pH 7.0, and the mixture was quickly submitted to detection by UV/Visible spectrophotometer, the absorbance change of this mixture within a time period of 5 min was monitored at the wavelength mentioned above. 6 different concentrations of the substrate ([S]) were tested to give 6 absorbance vs. time curves, each curve was used to calculate an initial hydrolysis rate (*v*_0_) according to the equation: *v*_0_ = ΔA/ΔTΔε, then all the *v*_0_ and [S] values were fitted into the Michaelis–Menten equation (Eq. 2) to give the maximum reaction rate (V_max_) and Michaelis constant (*K*_*M*_).

2 $${v}_{0}=\frac{{{\rm{V}}}_{{\rm{\max }}}[{\rm{S}}]}{{K}_{M}+[{\rm{S}}]}$$

Furthermore, the turnover number (*k*_*cat*_) was also obtained through the equation: *k*_*cat*_ = V_max_[E_0_], while [E_0_] is the initial concentration of enzyme.

#### Determination of pH profile for PenP WT and Glu166Tyr mutant

The assay was performed by monitoring initial velocities of nitrocefin hydrolysis at a range of substrate concentrations and pH conditions. The buffers used for the experiment were 50 mM sodium acetate (pH 5–6), 50 mM sodium phosphate (pH 6–7), 50 mM Tris (pH 7–9), 50 mM glycine (pH 9–10.5), 50 mM sodium bicarbonate (pH 11). Each buffer was supplemented with 150 mM NaCl. Initial velocity data were analyzed with GraphPad Prism 5 and fitted to the Michaelis-Menten equation. The pH dependence of *k*_*cat*_*/Km* was fitted to Eq. 3^30^.

3 $${{\rm{k}}}_{{\rm{obs}}}=\frac{{{\rm{k}}}_{{\rm{lim1}}}\times {10}^{{({\rm{pK}}}_{1}-{\rm{pH}})}+{{\rm{k}}}_{{\rm{lim2}}}}{1+{10}^{{({\rm{pK}}}_{1}-{\rm{pH}})}+{10}^{({\rm{pH}}-{{\rm{pK}}}_{2})}}$$

#### Crystallization and structure determination

The crystals of PenP Glu166Tyr were grown at 16 °C by the hanging drop vapor diffusion method. One μL of protein solution in a buffer of 20 mM Tris, 50 mM NaCl, pH 7.5 was mixed with 1 μL of reservoir buffer, which was composed of 0.1 M Tris pH 8.0, 22.5% PEG3350, 0.4 M ammonium acetate. Crystals appeared in 4–5 days and were harvested after they had grown to ∼100 μm in size. Cephaloridine was soaked into the crystals by incubating Glu166Tyr *apo* crystals in reservoir buffer containing 0.1 M cephaloridine for 7 minutes. To obtain the deacylation intermediates, the crystals of Glu166Tyr were soaked in 0.1 M cephaloridine-containing reservoir buffer first and then transferred to cephaloridine-free reservoir buffer for various time periods before mounted to the in-house Rigaku MicroMax^TM^−007HF X-ray machine for data collection. Diffraction data were collected at 100 K, integrated by iMosflm and scaled by the SCALA module in CCP4^31,32^. All the structures were solved by molecular replacement using the PHASER module in the CCP4i suite of programs with PenP wild-type structure (PDB ID: 4BLM) as search model^33^. The subsequent structural refinement was conducted using REFMAC module in CCP4^34^. Manual structure rebuilding was done using WINCOOT^35^. Data collection and refinement statistics are summarized in Table 2. The coordinates of all structures have been deposited to Protein Data Bank with the respective PDB ID listed in Table 2. The structure figures in Figs. 3–5 were prepared using the CCP4mg package in CCP4^36^.

*Classical MD* The initial conformation of Glu166Tyr was built upon the deacylation-3s structure (PDB ID: 5ZFT). The protonation states of the ionizable residues were determined at pH7 based on pKa calculations via both PROPKA^37^ and H++^38,39^ programs. If these two programs produce in-consistent predictions, we also take into account the local hydrogen bonding network. The partial charges of acylated CED were fitted with HF/6–31 G(d)^40^ calculations using the restrained electrostatic potential (RESP) module^41^ in the AmberTools package. The whole system was solvated into explicit TIP3P water molecules^42^ using a cubic box with a 12 Å buffer distance between the box wall and its nearest solute atom, and the Na^+^ ions were added to neutralize the charge. As a result, the whole system contains about 55,000–65,000 atoms. We then followed the same sophisticated protocol as in a previous study^14,43^ to conduct energy minimization and equilibration. In particular, we first performed a 2,500-step of steepest descent minimization followed by a 2500-cycle conjugate gradient minimization by restraining the protein and ligand (with restraint force constant of 50 kcal·mol^−1^·Å^−2^). We then performed a 150-ps NVT equilibration simulation (*T* = *10* *K*) followed by another NPT (*P* = *1* *atm*) equilibration simulation, during which the restrain force constant was gradually decreased to 25 kcal·mol^−1^·Å^−2^. Next, we performed a 250-ps temperature annealing NVT simulation (T was raised from 10 K to 300 K), during which we also reduced the restraint force constant to 10 kcal·mol^−1^·Å^−2^. Finally, we performed two sequential 150-ps NPT simulations to further reduced the restraint force constant to 1 kcal·mol^−1^·Å^−2^, and then finally to zero. Three independent production NPT (*T* = *300* *K* and *P* = *1* *atm*) simulations were then carried out for 10-ns with different initial velocities. In all MD simulations, long-range electrostatic interactions were treated with particle mash Ewald (PME) method^44,45^, and 12 Å cutoff was used for both PME short-range and van deer Waals (vdW) interactions. The Velocity-rescaling thermostat^46^ (with coupling constant of 0.1 ps^−1^), and the Parrinello-Rahman barostat^47^ (with the coupling constant of 1.0 ps^−1^) were adopted for temperature and pressure coupling respectively. All the MD simulations were performed using the AMBER12^48^ molecular dynamic package, and the Amber99SB-ILDN^49^ force field was employed.

*QM/MM* The initial structures for hybrid Quantum Mechanics/Molecular Mechanics (QM/MM) calculations were prepared based on a snapshot chosen from the 10-ns molecular dynamics simulation. The QM sub-system was treated by B3LYP^50–52^ functional with 6–31 G(d)^53,54^ basis set^55–57^, while all other atoms were described by the same molecular mechanical force field used in classical MD simulations. The QM/MM boundary was described by the improved pseudobond approach^58,59^. The prepared QM/MM systems were minimized and then employed to map out a minimal energy reaction path with reaction coordinate driving method^60^. All QM/MM minimization calculations were performed with the Q-Chem/Amber QM/MM interface^61,62^.The choice of QM sub-system is based on the proposed reaction schemes^63^. For the deacylation pathway in the “attack” conformation, QM sub-system contains acylated CED, deacylation water WAT_Nu, and side chains of Ser70, Tyr166, and Lys73. For the substrate assisted deacylation pathway in the “tilted” conformation, only acylated CED, deacylation water WAT_Nu, and side chains of Ser70 are included in the QM sub-system. The one dimensional minimal potential energy pathways (Figs. 6c and 7c) were mapped out using Dijkstra’s algorithm^64^ from the two dimensional potential energy surface for the deacylation start from “attack” and “titled” conformations respectively. In addition, to validate the convergence of our QM/MM minimization calculations, we constructed about multiple rounds of QM/MM path-scan calculations by Q-Chem/Amber interface, and the statistical errors for the potential energies of the stationary points along the reaction pathways were calculated.

## Supplementary information

Supplementary information.
