# Supplementary material for: The hydrolytic water molecule of Class A β-lactamase relies on the acyl-enzyme intermediate ES* for proper coordination and catalysis
Source: Sci Rep. 2020 Jun 23;10:10205. doi: 10.1038/s41598-020-66431-w (PMC7311446; doi:10.1038/s41598-020-66431-w)
Supplement: Supplementary file 1 — Supplementary information. [file 41598_2020_66431_MOESM1_ESM.docx]

**Supplemental Figure S1.** The critical structures along the reaction pathway starting from the “attack” conformation in our QM/MM calculations. INT: acyl adduct intermediate. TI: tetrahedron intermediate. PC: product complex. Figure is prepared using PyMOL2.3 (https://pymol.org/2/)


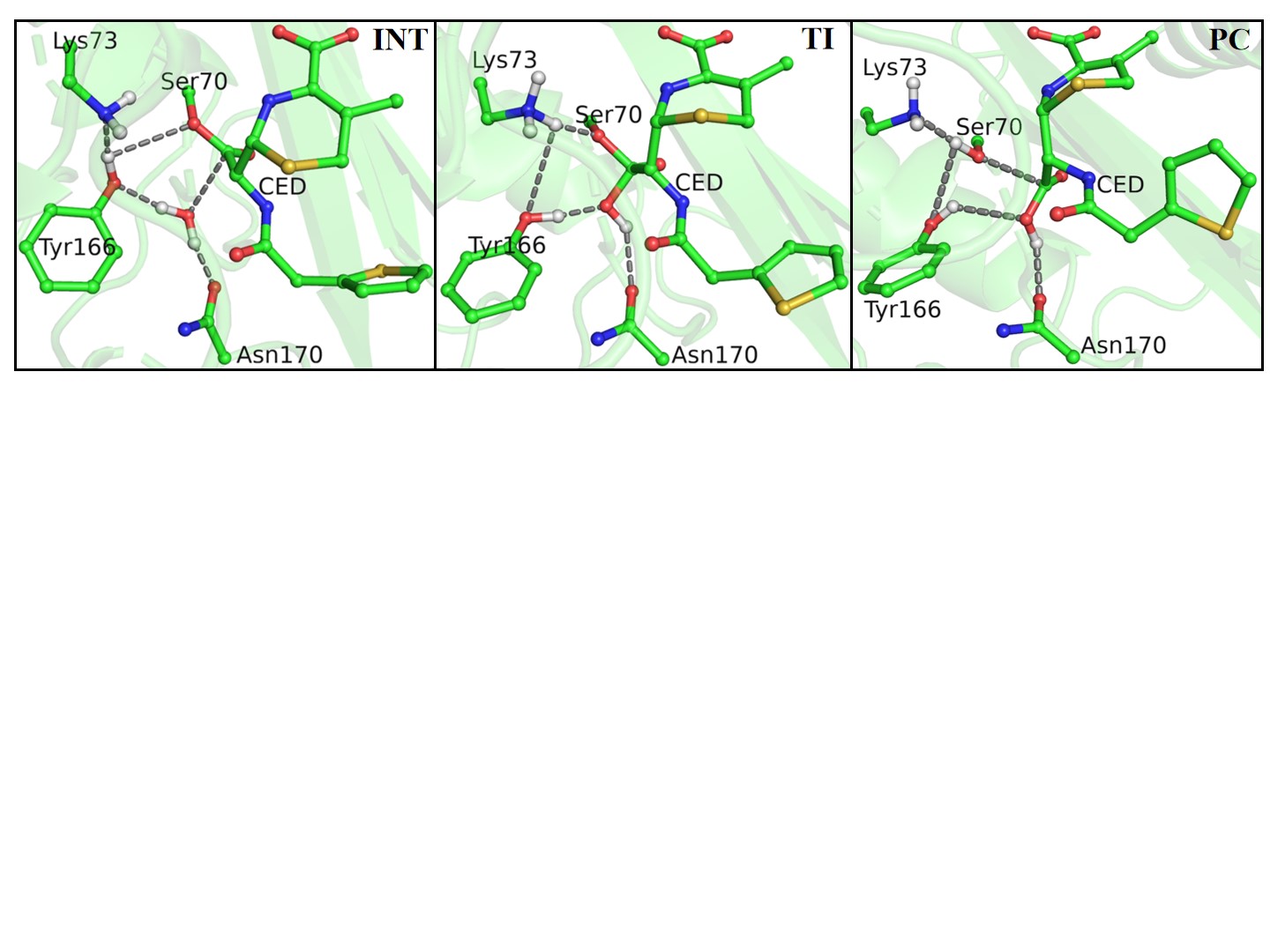


**Supplemental Figure S2.** The convergence test for the QM/MM minimizations by carrying out multiple rounds of QM/MM path-scan calculations for the deacylation of the “attack” (a) and “titled” (b) conformations.

**
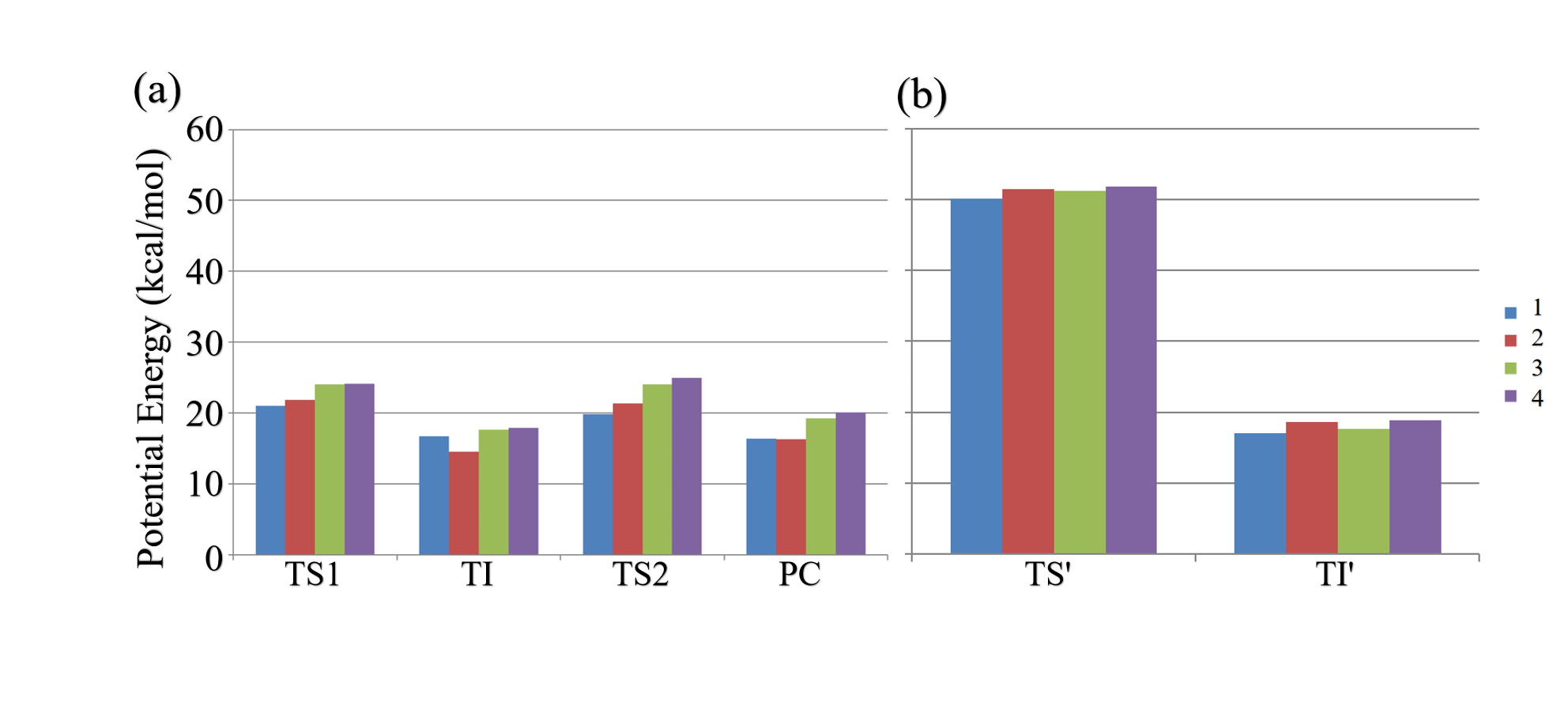
**
